# NADH-dependent biosensor in *Saccharomyces cerevisiae*: principle and validation at the single cell level

**DOI:** 10.1186/s13568-014-0081-4

**Published:** 2014-10-30

**Authors:** Jan Dines Knudsen, Magnus Carlquist, Marie Gorwa-Grauslund

**Affiliations:** 1 Division of Applied Microbiology, Department of Chemistry, Lund University, Lund, 221 00, SE, Sweden

**Keywords:** Saccharomyces cerevisiae, Redox balance, NADH biosensor, Single cell analysis

## Abstract

A reporter system was constructed to measure perturbations in the NADH/NAD^+^ co-factor balance in yeast, by using the green fluorescent protein gene under the control of the *GPD2* promoter that is induced under conditions of excess of NADH. High fluorescence levels were obtained in a glycerol 3-phosphate dehydrogenase double deletion strain (*gpd1Δgpd2Δ*), which is deficient in the ability to regenerate NAD^+^ via glycerol formation. The responsiveness of the reporter system to externally induced perturbations in NADH oxidation was also evaluated in the *gpd1Δgpd2Δ* strain background by addition of acetoin, as well as by introduction of a set of heterologous xylose reductases (XRs) having different selectivities for NADH. Addition of acetoin during cell proliferation under oxygen-limited conditions resulted in a more than 2-fold decrease in mean fluorescence intensity as compared to the control experiment. Strains carrying XRs with different selectivities for NADH could be distinguished at the single cell level, so that the XR with the highest selectivity for NADH displayed the lowest fluorescence. In conclusion, the designed system successfully allowed for monitoring perturbations in the cellular redox metabolism caused by environmental changes, or by heterologous gene expression. The reporter system displayed high resolution in distinguishing cytosolic NADH oxidation capacity and hence has potential to be used for high-throughput screening based on the fluorescence of single cells.

## 1 Introduction

The relative concentrations of NADH/NAD^+^ redox couple determine the thermodynamic feasibility of more than 100 reactions in cellular metabolism which places it at the core of the metabolic network of every organism including *Saccharomyces cerevisiae* (Förster et al. [2003]). Efficient tools to measure the impact of redox enzymes or environmental perturbations on the cellular NADH levels are therefore needed (Murray et al. [2011]). However quantification of redox couples in cells is complicated by several factors: the NADH and NAD^+^ are compartmentalized in *S. cerevisiae* and the cofactors are to a large extent in a protein bound form (Veech et al. [1969]; Bücher et al. [1972]). Fluorescence imaging enables the distinction between free and bound redox couples, but not between NADH and NADPH (Zhang et al. [2002]). In mammalian cells, coupled enzymatic reactions have been used to determine the ratio between the cofactors NADH/NAD^+^ (Williamson et al. [1967]; Theobald et al. [1997]). A similar approach has been applied for *S. cerevisiae* where mannitol-1-phosphate 5-dehydrogenase (M1PDH) was introduced and the cytosolic free NADH/NAD^+^ ratio was determined by the ratio between fructose-6-phosphate (F6P) and mannitol-1-phosphate (M1P) and the equilibrium constant for the reaction M1P + NAD ↔ F6P + NADH + H^+^ (Canelas et al. [2008]). The approaches are however often tedious and not applicable for high-throughput screening.

In *S. cerevisiae*, the cytosolic enzyme glycerol-3-phosphate dehydrogenase (GPD) 2 encoded by the *GPD2* gene plays a central role in redox metabolism. Under anaerobiosis, *GPD2* expression increases as the need for cytosolic reoxidation of NADH increases because Gpd2p acts as redox sink via the NADH-coupled reduction of dihydroxyacetone phosphate (DHAP) to glycerol 3-phosphate (G3P). G3P is further converted to glycerol (Eriksson et al. [1995]; Ansell et al. [1997]). The need for NADH reoxidation is thought to be linked, indirectly or indirectly, to the NADH/NAD^+^ ratio (Ansell et al. [1997]). Glycerol production can also be initiated by the isoenzyme Gpd1p encoded by *GPD1*. However Gpd1p and Gpd2p are expressed under different growth conditions, as illustrated by their different cellular localization (Valadi et al. [2004]). The physiological role of Gpd1p is also different from that Gpd2p as *GPD1* is induced under osmotic stress (Blomberg and Adler [1989]). Although the genes can complement each other’s functions, a double deletion renders the yeast cell osmosensitive as well as incapable of anaerobic growth, due to the inability to reoxidize cytosolic NADH arising from biomass formation (van Dijken and Scheffers [1986]; Nissen et al. [1997]).

We used this knowledge to design a GFP-based biosensor for cytosolic NADH/NAD^+^ levels by coupling the *GPD2* promoter (*GPD2*p) to the *yEGFP3* gene encoding yeast enhanced green fluorescent protein, an established yeast reporter of the GFP class 2 (Cormack et al. [1996]; Cormack et al. [1997]; Zacharias and Tsien [2005]). The applicability of fluorescent proteins as reporters has previously been demonstrated through the measurement of various user-specified cell properties. For example, GFP has been used for protein localization (Valadi et al. [2004]), intracellular pH measurement (Valkonen et al. [2013]), for monitoring cell growth mode and cell membrane robustness (Carlquist et al. [2012]). Dardalhon et al. ([2012]) also implemented a fluorescent glutathione redox reporter in *S. cerevisiae* using a yellow fluorescent protein sensor that reports on the nuclear and cytosolic glutathione redox state i.e the glutathione/glutathione disulfide (GSH/GSSG) state, which is NADPH dependent.

The functionality of the NADH/NAD^+^-ratio biosensor developed in this work was first evaluated by introducing it into *S. cerevisiae* strains with reduced NADH reoxidation capacity due to the deletion of Gpd1,2p encoding genes. The biosensor potential was then validated by evaluating the impact on cell fluorescence of acetoin addition and of the introduction of xylose reductases with different selectivities for NADH.

## 2 Methods

### 2.1 Strains

*S. cerevisiae* strains used in the study are summarized in Table 1. *Escherichia coli* NEB 5α (New England BioLabs, Ipswich, MA, USA) was used for subcloning. All strains were stored in 20% glycerol at -80°C. Yeast cells from freshly streaked YNB plates (6.7 g/L yeast nitrogen base without amino acids, 20 g/L glucose, 2% agar) were used for all cultivations.

**Table 1:** Plasmids and yeast strains used in the present study

**Strains and plasmids**	**Relevant features**	**Reference**
Plasmids		
YIplac211	*URA3*	(Gietz and Sugino [1988])
p424	*TRP1 Mumberg vector*	(Mumberg et al. [1995])
P425	*LEU2 Mumberg vector*	(Mumberg et al. [1995])
YEplacHXT	*URA3*	(Karhumaa et al. [2005])
pYGFP3	*URA3 ADH1p-yEGFP3-ADH1t*	(Cormack et al. [1997])
YIpJK01	*HIS3 GPD2p-yEGFP3-PGK1t*	This study
YEpJK01	*URA3 GPD2p-yEGFP3-PGK1t*	This study
YIpOB8	*URA3 TDH3p-XYL1-ADH1t PGK1p-XYL2-PGK1t*	(Bengtsson et al. [2009])
YIpDR7	*URA3 TDH3p-XYL1(N272D)-ADH1t PGK1p-XYL2-PGK1t*	(Runquist et al. [2010])
YIpCR01	*URA3 TDH3p-XYL1-ADH1t*	This study
YIpCR03	*URA3 TDH3p-XYL1 (N272D)-ADH1t*	This study
*S. cerevisiae* strains		
CEN.PK2-1C	*MATa ura3-52 trp1-289 leu2-3,112 his3Δ 1 MAL2-8C SUC2*	EUROSCARF
CEN.PK113-7D	*MATα, MAL2-8*^*c*^*SUC2*	EUROSCARF
TMB4120	CEN.PK2-1C YEpJK01 *trp1*::*TRP1 leu2*::*LEU2 his3::HIS3*	This study
TMB4121	CEN.PK2-1C YEpJK01 *gpd1*::*TRP1 leu2*::*LEU2 his3::HIS3*	This study
TMB4122	CEN.PK2-1C YEpJK01 *trp1*::*TRP1 gpd2*::*LEU2 his3::HIS3*	This study
TMB4123	CEN.PK2-1C YEpJK01 *gpd1*::*TRP1 gpd2*::*LEU2 his3::HIS3*	This study
TMB4132	CEN.PK2-1C *his3*::YipJK01 *trp1*::*TRP1 leu2*::*LEU2 ura3*^*-*^	This study
TMB4133	CEN.PK2-1C *his3*::YipJK01 *gpd1*::*TRP1 gpd2*:*LEU2 ura3*^*-*^	This study
TMB4140	TMB4132 *ura3*::YIplac211	This study
TMB4141	TMB4132 *ura3*::YIpCR01	This study
TMB4143	TMB4132 *ura3*::YIpCR03	This study
TMB4144	TMB4133 *ura3*::YIplac211	This study
TMB4145	TMB4133 *ura3*::YIpCR01	This study
TMB4147	TMB4133 *ura3*::YIpCR03	This study

### 2.2 Molecular biology methods

Plasmid DNA was prepared using the GeneJET Plasmid Miniprep Kit (Thermo Scientific, USA). Restriction and modification enzymes as well as T4 DNA ligase were obtained from the same manufacturer. The QIAquick gel extraction kit (QIagen, Hilden, Germany) was used for DNA extractions from agarose. All nucleotides were ordered at Eurofins (Germany). All genetic constructs were checked by sequencing (Eurofins, Germany). The primers used in the study are listed in Table 2.

**Table 2:** Primers used in the present study

**Name**	**Description**	**Sequence**
TRP1_gpd1Over_f	Forward primer for amplification of *TRP1* (p424 as template) with *GPD1* overhangs	TCCACAAACACAAATATTGATAATATAAAGAACGACATTACTATATATATAATATAG
TRP1_gpd1Over_r	Reverse primer for amplification of *TRP1* (p424 as template) with *GPD1* overhangs	AGTATGATATGTTATCTTTCTCCAATAAATAGGCAAGTGCACAAACAATAC
TRP1_gpd1_veri_f	Amplification of the 5′ region of *GPD1* (CEN.PK113-7D as template)	TTCCATTCACATATCGTCTTTGG
	+ Verification of *GPD1* deletions	
5-OEGpd1_r	Amplification of the 5′ region of *GPD1* (CEN.PK113-7D as template)	TATATTATCAATATTTGTGTTTGTGG
3-OEGpd1_f	Amplification of the 3′ region of *GPD1* (CEN.PK113-7D as template)	TTTATTGGAGAAAGATAACATATCATAC
3-OEGpd1_r	Amplification of the 3′ region of *GPD1* (CEN.PK113-7D as template)	ATTTTCTTAGGACGCCGCAAAATATC
TRP1_gpd1_veri_r	Verification of *GPD1* deletions	GAGGAACTCTTGGTATTCTTGCC
LEU2_gpd2Over_f	Forward primer for amplification of *LEU2* (p425 as template) with *GPD2* overhangs	TTCCTTTTCCTTCGCTCCCCTTCCTTATCATCGACTACGTCGTAAGGCCG
LEU2_gpd2Over_r	Forward primer for amplification of *LEU2* (p425 as template) with *GPD2* overhangs	GATCAGAGGGGGAGGGGGGGGGAGAGTGTCGAGGAGAACTTCTAGTATATC
LEU2_gpd2OO_f	Forward primer for nested PCR for adding homology regions to the *GPD2* deletion casette	GTATTTTGGTAGATTCAATTCTCTTTCCCTTTCCTTTTCCTTCGCTCCC
LEU2_gpd2OO_r	Reverse primer for nested PCR for adding homology regions to the *GPD2* deletion casette	AAATTGGTTGGGGGAAAAAGAGGCAACAGGAAAGATCAGAGGGGGAGGG
LEU2_gpd2_veri_f	Verification of *GPD2* deletions	CGTGTATCTTCTAAGATTCAGTC
LEU2_gpd2_veri_r	Verification of *GPD2* deletions	CTAATGGCTCAACGTGATAAGG
GPD2p_XbaI_r	Cloning of *GPD2* from CEN.PK113-7D	CTTCTAGATTGATAAGGAAGGGGAG
GPD2p_SacI_f	Cloning of *GPD2* from CEN.PK113-7D	TCGAGCTCCGCAATGTTTCGTTGG
yEGFP_XbaI_f	Cloning of *yEGFP3* from pyEGFP3	AATCTAGAAGCATTAAAAAATGTCTAAAGGTGAAG
yEGFP_PstI_r	Cloning of *yEGFP3* from pyEGFP3	TTCCTGCAGAGTTATTTGTACAATTCATCCATAC
HIS3_flank_f	Cloning of *HIS*3 - Forward primer	CCAGGTATCGTTTGAACACGG
HIS3_flank_r	Cloning of *HIS*3 - Reverse primer	GCTCAGTTCAGCCATAATATG
LEU2_flank_f	Cloning of *LEU2* - Forward primer	GGATAATTATACTCTATTTCTCAAC
LEU2_r	Cloning of *LEU2* - Reverse primer	TTAAGCAAGGATTTTCTTAAC
TRP1_f	Cloning of *TRP1* - Forward primer	ATGTCTGTTATTAATTTCAC
TRP1_r	Cloning of *TRP1-* Reverse primer	CTATTTCTTAGCATTTTTG
YIplac211_f	Cloning of *GPD2*p-*yEGFP3*-*PGK*1t	TTTATCTTCGTTTCCTGC
YIplac211_r	Cloning of *GPD2*p-*yEGFP3*-*PGK1*t	AAAACTGTATTATAAGTAAATG
HIS3_O_f	Cloning of *HIS3*- expression cassette, for change of auxotrophic marker.	TTATAATACAGTTTTCCAGGTATCGTTTGAACACGG
HIS3_O_r	Cloning of *HIS3*-expression cassette, for change of auxotrophic marker.	GGAAACGAAGATAAATCGCTCAGTTCAGCCATAATATG

Competent cells of *E. coli* NEB 5α were prepared and transformed by the method of Inoue et al. ([1990]) and yeast transformations were performed using the lithium acetate method (Gietz and Woods [2002]). The cells were streaked out on selective medium afterwards. *E. coli* transformants were selected on Lysogene Broth (LB) plates (Sambrock and Russell [2001]) with 50 μg/ml ampicillin (IBI Shelton Scientific, Shelton, USA). *S. cerevisiae* transformants were selected on YNB plates supplemented with the requested amino acids or base.

### 2.3 Construction of deletion and insertion cassettes

The *GPD1* deletion cassette was constructed by joining three PCR fragments through overlap extension PCR (Sandström et al. [2012]). A nucleotide fragment homologous to the upstream region of *GPD1* was PCR amplified from genomic DNA of CEN.PK113-7D strain using the primer couple TRP1*_gpd1_veri_f*, 5-OEGpd1_r. The promoter, ORF and terminator of *TRP1* were PCR amplified from a Mumberg p424 vector using the primers TRP1_gpd1Over_f, TRP1_gpd1Over_r. The downstream region of *GPD1* was PCR amplified from genomic CEN.PK113-7D DNA using the primer couple 3-OEGpd1_f, 3-OEGpd1_r. The three fragments were all purified before being combined into one fragment using the nucleotides TRP1_gpd1Over_f, 3-OEGpd1_r.

The *GPD2* deletion cassette was constructed by nested PCR on a PCR fragment containing the promoter, ORF and terminator of *LEU2.* The template fragment was constructed using the primers LEU2_gpd2Over_f, LEU2_gpd2Over_r. The fragment was purified and then used as template using the primers LEU2_gpd2OO_f, LEU2_gpd2OO_r. The added regions contained around 50 bp regions homologous to the upstream and downstream region of *GPD2,* respectively.

Expression cassettes for curing tryptophan, leucine and histidine auxotrophies were constructed using CEN.PK113-7D as a template and corresponding primer couples (TRP1_f, TRP1_r, LEU2_flank_f, LEU2_r and HIS3_flank_f, HIS3_flank_r, respectively) to PCR amplify the promoter, gene and terminator of the respective genes.

### 2.4 Plasmid construction

Plasmid YEpJK01was constructed by ligation of three DNA fragments using T4 DNA ligase: 1) A 1.4 kb region directly upstream of the *GPD2* ORF that was PCR amplified from CEN.PK113-7D genomic DNA using the primers GPD2p_XbaI_r and GPD2p_SacI_f, digested with *Sac*I and *Xba*I and purified, 2). The *yEGFP3* fragment that had been PCR amplified from pYGFP3, using the primers yEGFP_XbaI_f and yEGFP_PstI_r, digested with *Xba*I and *Pst*I and finally purified, and 3) YEplacHXT digested with *Sac*I and *Pst*I. The ligation mix was used to transform *E. coli*.

Plasmid YIpJK01was constructed by excision of the *GPD2p-yEGFP3-PGK1t* cassette from YEpJK01 plasmid using the restriction enzymes *Sph*I and *Sac*I. The fragment was purified from agarose gel and ligated to YIplac211 plasmid that was previously digested with the same restriction enzymes. The auxotrophic marker of the resulting plasmid was changed from *URA*3 to *HIS3* by PCR amplifying the entire plasmid except for the *URA3* region. The amplification was done using the primers YIplac211_f, YIplac211_r. The fragment was then recombined with a PCR fragment containing a *HIS3* expression cassette using the *In-Fusion® HD Cloning Kit*. The *HIS3* cassette was obtained from CEN.PK113-7D genomic DNA using the nucleotides HIS3_O_f, HIS3_O_r. The primers were designed according to the *In-Fusion® HD Cloning Kit* guidelines*.* The entire YEpJK01 plasmid was transformed into *E. coli*.

For the construction of YIpCRO1 and YIpCRO3, the *XR*-expression cassettes were excised from YIpOB8 and YIpDR7, respectively, using *Hind*III. The vector YIplac211 was cleaved using the same enzyme. The relevant fragments were isolated and purified. Ligation of YIplac211 and *TDH3p-XYL1-ADH1t* yielded YIpCR01. Ligation of YIplac211 and *TDH3p-XYL1(N272D)-ADH1t* yielded YIpCR03.

### 2.5 Construction of yeast strains

TMB4120, TMB4121, TMB4122, TMB4223 (Table 1) were all constructed using CEN.PK2-1C as parental strain. The strains were made prototrophic by transforming sequentially the CEN.PK2-1C strain with (i) the required expression cassettes for *LEU2*, *TRP1* or *HIS3*, (ii) the *GPD1* and/or *GPD2* deletion cassette and (iii) YEpJK01plasmid.

TMB4132 and TMB4133 were constructed by transformation of CEN.PK2-1C with expression cassettes for *TRP1* and *LEU2* or the *GPD1 (TRP1)* and *GPD2 (LEU2)* deletions cassettes. Finally the strains were transformed with YIpJK01 that was linearized with *Nhe*I.

TMB4140, TMB4141, TMB4143, TMB4144, TMB4145 and TMB4147 were constructed by transforming TMB4132 or TMB4133 with YIplac211, YIpCR01 or YIpCR03 respectively. All vectors were linearized with *Apa*I prior to transformation.

Single gene deletions were verified by PCR amplification whereas double deletions were verified by PCR screening and enzymatic assay (described below). *GPD1* deletions were PCR verified using the nucleotide couple TRP1_gpd1_veri_f and TRP1_gpd1_veri_r. *GPD2* deletions were verified using the nucleotide couples LEU2_gpd2_veri_f and LEU2_gpd2_veri_r

### 2.6 Cell cultivation

For the initial Flourescence intensity (FI) measurements, single colony cells were taken from YNB plates not older than 2 weeks and inoculated in 250 ml shake flasks containing 25 ml YNB medium buffered to pH 5.5 with potassium hydrogen phthalate and complemented with 2% glucose. The cells were grown overnight at 180 rpm and 30°C.

For the FI measurement of XR strains, pre-cultivations and cultivations were done in YNB medium buffered to pH 5.5 with potassium hydrogen phthalate and complemented with 2% galactose and 2% xylose. The cells from the pre-cultivation step were washed with sterile water before inoculation. Oxygen-limited cultures were performed in 65 mL flasks with 45 mL medium and ergosterol and Tween-80 were added to the medium at a final concentration of 0.42 g/L and 0.01 g/L respectively (Andreasen and Stier [1953], [1954]). Bottles were closed with rubber stoppers and cannulas were used for gas outlet and sampling, thus minimizing oxygen transfer. Cultures were stirred gently with magnetic stirrers and incubated at 30°C. Fermentation was performed in biological duplicates. Substrate consumption and product formation values were determined from samples withdrawn during 72 h of fermentation.

### 2.7 Analyses

Samples for OD_600_ were analyzed directly while samples for HPLC were kept at −20°C. Growth was monitored by measuring OD at 620 nm.

Xylose, galactose, glycerol, ethanol and xylitol were separated and quantified by high-pressure liquid chromatography (HPLC) (Waters, Milford, MA, USA). The compounds were separated with a HPX-87P (Bio-Rad, Hercules, CA, USA) ion exchange column). Separation was performed at 80°C, with H_2_O as mobile phase at a flow rate of 0.6 mL/min. A refractive index detector (RID-6A; Shimadzu, Kyoto, Japan) was used for quantification.

A BD Accuri^TM^ C6 (Becton-Dickinson, NJ, USA) was used for the evaluation of the redox biosensor in the different deletions strains. Fresh cells were used. Samples for flow cytometry were centrifuged for 1 min at 3000g and 4°C, and resuspended in saline solution. The impact of the reporter gene copy number on fluorescence heterogeneity was evaluated on A BD FACSort (Becton-Dickinson, NJ, USA) flow cytometer. Fresh cells were harvested at the late exponential phase. Evaluation of the biosensor responsiveness to redox perturbations by heterologous expression of NADH-dependent oxidoreductases was performed with a BD FACSAria III (Becton-Dickinson, NJ, USA) flow cytometer. Samples were frozen in phosphate buffer, pH 7, 25% glycerol at -80°C before being thawed and analyzed. Events were recorded with a rate of approximately 1,000 events per second. Excitation wavelength for the lasers used was 488 nm. Fluorescence emission levels were measured using a band pass filter at 530/30 nm (FI). Processing and analysis of flow cytometry raw data was performed by using MatLab®R2010b (The MathWorks,Inc., Natick, MA, USA). The measurement files, exported as fcs files by the flow cytometer FACSAria III, were imported into MATLAB, using a “fcs data reader” routine (by L. Balkay, University of Debrecen, Hungary), available on MATLAB W File Exchange website.

## 3 Results

### 3.1 Construction and evaluation of the redox biosensor

In order to construct a biosensor that responds to cytosolic NADH/NAD^+^ ratio, a 1.4 kb region upstream of the *GPD2* open reading frame that includes the whole *GPD2* promoter region (Eriksson et al. [1995]), was cloned 18 bp upstream of the start codon of the *yEGFP3* gene (Cormack et al. [1997]). The resulting *GPD2*p-*yEGFP3* expression cassette was introduced in the multicopy vector YEplacHXT generating plasmid YEpJK01. The YEpJK01 plasmid was then introduced into a control laboratory strain as well as in redox engineered strains carrying either single or double deletion of *GPD1* and *GPD2* genes, thereby generating strains TMB4120 (control), TMB4121 (*gpd1Δ*), TMB4122 (*gpd2Δ*) and TMB4123 (*gpd1Δ gpd2Δ*).

**Figure 1:**
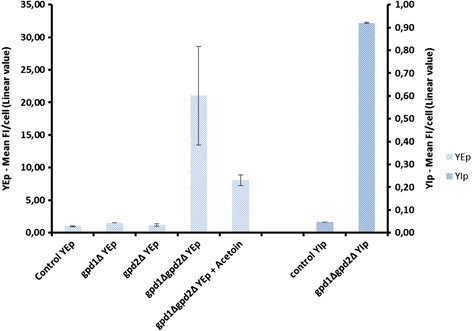
**Mean fluorescence intensities (FI) for the different strains (wild type,*gpd1Δ, gpd2Δ and gpd1Δgpd2Δ*) carrying either the multicopy (YEp)- or the integrative (YIp)- based GFP reporter.** Fluorescence intensities are given in linear values. All values are normalized to the control strain TMB4120 carrying YEp-based GFP reporter. Acetoin was added at a concentration of 0.5 g/L.

Strains were cultivated in batch mode under oxygen-limited conditions for 5 hours in defined mineral medium with glucose as sole carbon source, after which the FI was measured (Figure 1). At this time point, cells were in exponential growth phase and could be considered as being in pseudo-steady state with regards to regulation of the glucose catabolic pathways. All redox engineered strain had higher FI than the control strain TMB4120. However the double deletion strain TMB4123 (*gpd1Δ gpd2Δ*) had the highest FI (21.5 fold higher than the control), whereas each single deletion had moderate increase in FI (1.5 and 1.2 fold for *gpd1Δ* and *gpd2Δ*, respectively) (Figure 1). To further evaluate the biosensor responsiveness to perturbations in NADH/NAD^+^ ratio, the FI of TMB4123 (*gpd1Δ gpd2Δ*) was measured after addition of acetoin (3-hydroxy-2-butanone) during respiro-fermentative growth under oxygen-limited conditions. Acetoin is an alternative redox sink to the glycerol shunt/pathway through its NADH-dependent conversion to 2,3-butanediol (Bruinenberg et al. [1983]; Björkqvist et al. [1997]). The reaction is catalyzed by a NADH-dependent 2,3-butanediol dehydrogenase encoded by *BDH1* (Gonzalez et al. [2000]). Indeed the addition of acetoin led to reduced FI as the relative fluorescence decreased from 21.5 to 8.0 (Figure 1). Altogether our results demonstrated that the constructed biosensor reported on the cells capacity to oxidize NADH, and more generally on the NAD(H) cofactor balance.

### 3.2 Impact of the reporter gene copy number on fluorescence intensity and heterogeneity

**Figure 2:**
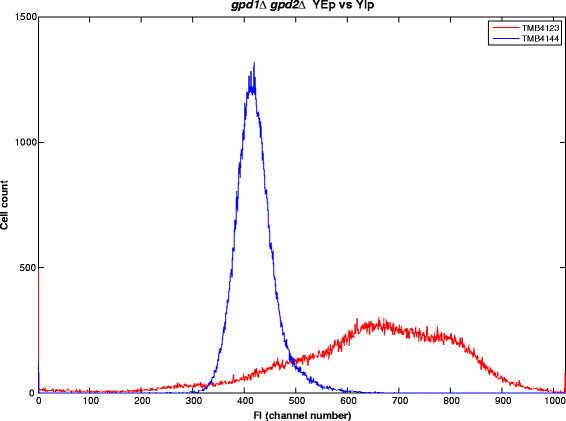
**Histograms of the fluorescence intensity of strains TMB4123 (*gpd1Δgpd2Δ*** **YEp-GFP, “red dash”) and TMB4144 (*gpd1Δgpd2Δ*** **YIp-GFP, “blue dash”) that were grown aerobically on glucose.**

To investigate the impact of the copy number of the reporter gene *yEGFP3* and only relate the expression level to the transcription step, the reporter cassette was also transferred to an integrative vector (YIp) and introduced into the same control and *gpd1Δ gpd2Δ* strain backgrounds, generating strains TMB4140 (control) and TMB4144 (*gpd1Δ gpd2Δ*). Strains were cultivated in shake flasks with YNB media and 2% glucose and FI was recorded. Similar trend was observed as for the YEp based reporter constructs. But the FI levels were lower, with strain TMB4140 (control- YIp) only emitting 4% of the FI signal of TMB4120 (control- YEp). However, a clear difference in FI was again observed between the wild type and the double mutant, as TMB4144 (*gpd1Δ gpd2Δ*- YIp) displayed 21.5 fold higher FI than its corresponding control TMB 4140 (Figure 1). Also the heterogeneity of the fluorescence signal that can be measured by the standard deviation of the population was lower in the YIp system (Figure 2). Standard deviations of 44 was measured in TMB 4144 (*gpd1Δ gpd2Δ*- YIp) whereas a standard deviation of 172 was obtained in TMB4123 (*gpd1Δ gpd2Δ*- YEp). Thus, to reduce the influence of gene copy number and plasmid replication kinetics on FI output in the population, further experiments were performed using the YIp based sensor.

### 3.3 Evaluation of the biosensor responsiveness to redox perturbations by heterologous expression of NADH-dependent oxidoreductases

The potential of the *GPD2*p-*yEGFP3* redox biosensor to differentiate between reductases having different selectivities for NADH was also tested. For this purpose, two NAD(P)H-dependent xylose reductases (XRs) that reduces xylose to xylitol using NADPH and NADH as cofactor were utilized: *Pichia stipitis* XR (wtXR) and the corresponding N272D mutant (mutXR) that display NADH/NADPH selectivity ratios of 0.04 and 0.80, respectively (Runquist et al. [2010]). Both the non-engineered and the *gpd1Δ gpd2Δ* yeast backgrounds were chosen as hosts as they represent the two redox extremes i.e. the strain with the best ability to reoxidize cytosolic NADH and the one with the poorest. The YIp-based reporter and XR constructs were introduced in these strains generating strains TMB4140 (control, no XR), TMB4141 (control, wtXR), TMB4143 (control, mutXR), TMB4144 (*gpd1Δgpd2Δ,* no XR), TMB4145 (*gpd1Δgpd2Δ,* wtXR) and TMB4147 (*gpd1Δgpd2Δ,* mutXR).

**Figure 3:**
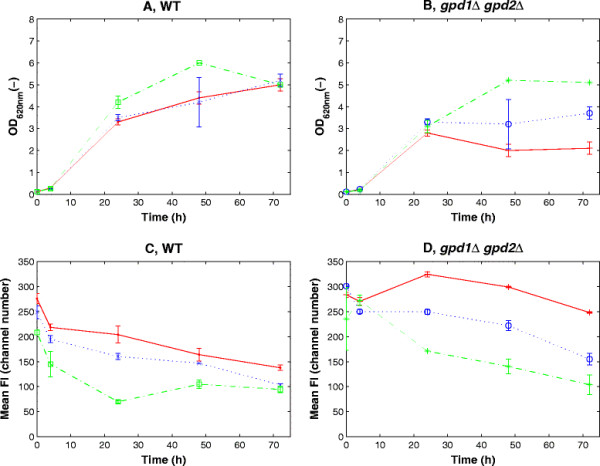
**Optical density measured at 620 nm (A, B) and mean fluorescence intensity (C, D) over time for the control background (A, C) or the*gpd1Δgpd2Δ*background (B, D) carrying no XR, wtXR or mutXR.** Legend: TMB4140 (control, no XR “red dot”), TMB4141 (control, wtXR “blue x”), TMB4143 (control, mutXR(green square)), TMB4144 (*gpd1Δgpd2Δ*, no XR “red plus”)), TMB4145 (*gpd1Δgpd2Δ*, wtXR “blue o”)), TMB4147 (*gpd1Δgpd2Δ*, mutXR “green asterisk”).

**Figure 4:**
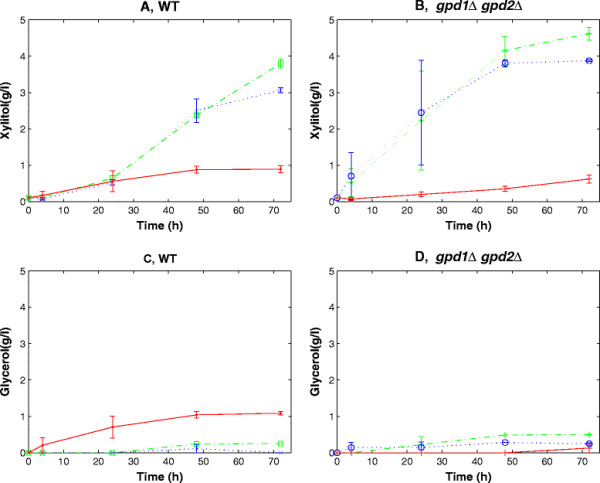
**Extracellular xylitol (A, B) and glycerol (C, D) concentrations (in g/L) over time for the control background (A, C) or the*gpd1Δgpd2Δ*background (B, D) carrying no XR, wtXR or mutXR.** Legend: TMB4140 (control, no XR “red dot”), TMB4141 (control, wtXR “blue x”), TMB4143 (control, mutXR(green square)), TMB4144 (*gpd1Δgpd2Δ*, no XR “red plus”), TMB4145 (*gpd1Δgpd2Δ*, wtXR “blue o”), TMB4147 (*gpd1Δgpd2Δ*, mutXR “green asterisk”).

Strains were cultivated under oxygen-limited conditions in sealed vials containing minimal medium with galactose as the carbon source and xylose as the substrate for the XR-catalysed reduction. Galactose was used because *S. cerevisiae* has a specific galactose uptake system, Gal2p (Cirillo [1968]) that also transports xylose, and whose expression benefits xylose utilization (Johnston [1987]; Hamacher et al. [2002]). So, even though the yeast does not metabolize galactose as fast as glucose, the use of galactose allows the cell to have co-consumption of hexose and xylose (Bro et al. [2005]; Garcia Sanchez et al. [2010]). The growth profiles of the different strains are shown in Figures 3A and B. During the first 24 hours, *gpd1Δ gpd2Δ* strains reached an OD of around 3, which was only slightly lower than the corresponding control strains. During the next 48 hours, however, TMB4147 strain (*gpd1Δ gpd2Δ,* mutXR) continued growing and reached an OD of 5.2 whereas strain TMB4145 (*gpd1Δ gpd2Δ,* wtXR) only reached OD 3.7 while the OD of strain TMB4144 (*gpd1Δ gpd2Δ*, no XR) decreased to 2 (Figure 3B). Also TMB4147 strain carrying the XR with the highest selectivity for NADH (mutXR) almost reached the same OD as the corresponding control strain TMB4143 (control, mutXR) (Figures 3A, B), which was not the case for the other strains carrying either no XR or wtXR. Improved NADH reoxidisation was expected to be coupled to the NADH-dependent xylose reduction to xylitol. Indeed high xylitol production, between 3 and 5 g/L, was measured during the first 48 hours of cultivations in all strains harboring a heterologous XR (Figure 4). Also the deletion strains produced xylitol faster, and in larger quantities than the control strains. Within the first 48 hours, there was no significant difference in xylitol production between strains carrying wtXR and mutXR, neither in the control strain background (Figure 4A) nor in the *gpd1Δ gpd2Δ* background (Figure 4B). However, the strains carrying mutXR reached slightly higher xylitol concentrations at the end of the cultivations. Xylitol was also produced in strains lacking heterologous XR, but to a significantly lower degree (<0.8 g/L after 70h). Xylitol may actually arise from the NADPH-dependent conversion of xylose by endogenous NADPH-dependent XR, such as the one encoded by *GRE3* gene (Träff et al. [2002]). In the case of XR-carrying strains, xylitol may also originate from the NADPH-catalysed reaction of the introduced XR. Glycerol production was observed for the double deletion strains carrying the control or mutated XR (Figure 4C, D), which may be explained by the fact that *P. stipitis* XR can use DHAP as substrate, which leads to glycerol formation (Jeppsson et al. [2003]).

Both the control and the *gpd1Δ gpd2Δ* strains showed the highest fluorescence at the beginning of the cultivation, followed by a gradual decrease until the end of fermentation. An inverse correlation was obtained between growth (Figure 3A, B) and the mean FIs (Figure 3C, D), with the best growing strains having the lowest fluorescence both in the control and the *gpd1Δ gpd2Δ* backgrounds. The difference in fluorescence was however less pronounced between the control strains than between the *gpd1Δ gpd2Δ* strains, which can be explained by the limited redox imbalance in the control strains.

**Figure 5:**
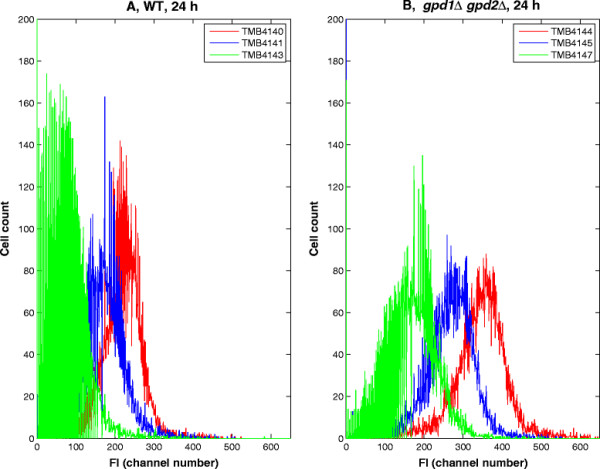
**Fluorescence plots after 24 hours of growth. (A)** Histograms of the FI of the control strains carrying no XR “red dash”, wtXR “blue dash” or mutXR “green dash”). **(B)** Histogram of the FI of the *gpd1Δ gpd2Δ* strains carrying no XR (red dash), wtXR (blue dash) or mutXR (green dash). Legend TMB4140: control, no XR; TMB4141: control, wtXR; TMB4143: control, mutXR; TMB4144: *gpd1Δgpd2Δ*, no XR;TMB4145: *gpd1Δgpd2Δ*, wtXR; TMB4147:*gpd1Δgpd2Δ*, mutXR.

The population histogram, i.e. the amount of single cells displaying a given FI, also clearly showed differences between the strains (Figures 5A, B). After 24 hours, the different populations displayed significantly different FIs, and were thus easily distinguished in an overlay plot. This was the case both in the control (Figure 5A) and in the *gpd1Δ gpd2Δ* (Figure 5B) strains, with mean FIs given in channel number units of 204 (control, no XR), 160 (control, wtXR), 70 (control, mutXR) or 325 (*gpd1Δ gpd2Δ*, no XR), 250 (*gpd1Δ gpd2Δ*, wtXR) and 171 (*gpd1Δ gpd2Δ*, mutXR), respectively. Some overlap between strains was observed in both strain backgrounds, but it was more pronounced for the wild type strains than for the deletions strains

## 4 Discussion

Microbial populations exist in dynamic equilibrium with constantly changing conditions, which requires fast and fluid regulatory networks for optimizing cell functions. Among these, an accurate metabolic response is fundamental for the cell to maintain redox homeostasis. In *S. cerevisiae*, it is well known that a skewed ratio of cytosolic NADH/NAD^+^ can be compensated by glycerol production regulated by a fine-tuned expression of the *GPD2* gene (Ansell et al. [1997]; Björkqvist et al. [1997]). This served as basis for the construction of our *in vivo* redox state reporter that enables green fluorescence to be used as an indicator of the cells ability to reoxidize NADH, *i.e.* to the NADH/NAD^+^ ratio of the cell. This is, to our knowledge, the first biosensor to use the *GPD2* promoter as sensor for the cytosolic NADH/NAD^+^ cofactor balance.

By placing a *GFP* gene downstream of the *GPD2* promoter, a direct relationship between green fluorescence intensity (FI) and glycerol formation or NADH accumulation was observed. In strains carrying double deletion of the *GPD* genes, the FI was significantly higher (21.5-fold) than for the control strain, indicating a strong activation of the *GPD2* promoter, and thereby verifying the lack of other enzymes to counteract the lacking NADH oxidation functionality. In parallel, FIs were in the same order of magnitude in single *GPD* deletion strain and in the control strain, also confirming the previously observed compensatory pattern between Gpdp isoenzymes in order to maintain NAD^+^ regeneration via glycerol production (Ansell et al. [1997]). The FI signal of the *gpd1*Δ strain was also slightly higher than the FI of the *gpd2*Δ strain and the control strain: 25% and 50%, respectively, as expression of the *GDP2*, gene whose promoter is used in the constructed system, should be higher in the *gpd1*Δ strain, as a consequence of *GPD2* compensating for the lacking *GPD1* functionality. Finally the high responsiveness of the constructed reporter supported the hypothesis that *GPD2* expression was directly connected to the cell need to oxidize NADH via glycerol formation. It cannot be excluded that FI may be correlated to more than the need for NADH oxidation via glycerol formation. Growth rate and the capacity to re-oxidise NADH are closely related and thus FI will always be indirectly correlated to growth rate. As an example of this, the rescued growth rate of the *gpd1Δgpd2Δ* deletion strain by increased NADH oxidation led to significantly lower FI. However the oxygen concentration, which reflects the cells ability to replace the need for glycerol formation by aerobic respiration, was probably a more important parameter in that strain. Cultivating cells under oxygen-limited conditions could also have had implications for the FI measurements as fluorescent proteins require oxygen to mature. However, GFP expression in *S. cerevisiae* could previously be measured in cells that were cultivated under strict anaerobic conditions, most probably due to exposure to oxygen during the aerobic handling of samples. (Hebisch et al. [2013]) previously demonstrated that the average maturation time in *E. coli* for GFP is around 6 min, which is enough time for GFP to mature during the preparation for the FI measurement in the present study.

There was a clear difference between placing the reporter cassette on a multicopy plasmid or on a plasmid that gets integrated in the chromosome. Copy number per cell varies between 50 and 200 when using YEp vectors as opposed to one when using YIp vectors (Romanos et al. [1992]). So placing the GFP-based reporter construct on YEp plasmids led to a significantly higher FI than when using YIp-based construct. However, more defined fluorescence peaks were obtained when using the YIp system as opposed to the YEp system. This is in accordance with previous findings illustrating the stability issues of YEp-based systems compared to YIp-based systems (Ishii et al. [2009]) and indicates lower degree of cell population heterogeneity in chromosomally integrated biosensors as compared to the YEp-based systems, which may later facilitate the distinction between any underlying subpopulations. Promoters can also have different levels of noise in the respective gene expression (Blake et al. [2003]), which can lead to a higher heterogeneity of the signal. The low degree of heterogeneity in the YIp-based system further stresses the suitability of the *GPD2* promoter for analysis of populations at the single cell level.

A 2-fold lowering of the GFP levels was observed when acetoin was added to *GPD* double deletion strains, which can be attributed to the induced NADH-dependent reduction of acetoin (Gonzalez et al. [2000]). Acetoin or acetaldehyde, that is reduced to ethanol with the concurrent oxidation of NADH, have previously been used to demonstrate the lack of alternative cytosolic reductive reactions in double deletion strain (Lidén et al. [1996]; Ansell et al. [1997]). In these studies, the involved oxidation reactions were efficient enough to recover sufficient redox balance for the cells to start growing. By monitoring FI instead, we wanted to investigate whether the constructed biosensor could monitor less drastic changes in the redox balance. To validate this hypothesis, we introduced XR enzymes with slightly different catalytic activity for NADH oxidation, namely two xylose reductases having NADH/NADPH selectivity ratios of 0.04 and 0.80 respectively (Runquist et al. [2010]). As expected, the biggest difference in growth was observed in the double deletion strains between the control and the strain carrying the mutXR, since more carbon was made available for growth when xylitol formation from xylose was used as redox sink instead of glycerol formation from glucose. This is also part of the explanation for the lower glycerol formation in the TMB4141 and TMB4143 as opposed to the TMB4140, as the cells can produce xylitol as a redox sink in addition to glycerol. A higher xylitol production could have been expected for the mutXR than wtXR in the *gpd1Δgpd2Δ* strain, if only considering the NADH-dependent activity. However wtXR also has a significant activity with NADPH as a cofactor (Rizzi et al. [1988]), which led to a non-negligible NADH-independent xylitol production. The maximum difference in OD was 2.5-fold after 48 hours of growth whereas the difference between the control strain and the strain carrying the wtXR was only 1.5 fold. So, in theory, the reductases could be distinguishable by repeating batch cultivations and using growth as selection. Assuming that after 48 hours one cell of TMB4144 (double deletion mutXR) grows to 2.5 times the cell density of the TMB4140 (double deletion no XR), it would take 27 batch cultivations of 48 hours each before one TMB4144 cell would outgrow 10^9^ TMB4140 cells by a factor of 1:100 (according to the following calculation: (1/2.5)^27^∙10^9^ ≈ 0.01). Using fluorescence instead, the difference was clearly visible after a few hours. Increased xylitol formation and lower fluorescence were observed in strains carrying the wtXR and the mutXR. However, a clear difference in FIs was observed between the two strains whereas differences in xylitol levels were not that significant. The results, although qualitative, indicate that fluorescence was the more sensitive and faster method to discriminate between the two XR enzymes and their difference in NADH-catalyzed xylitol production and consequently their ability to affect the cytosolic NADH/NAD^+^ ratio. It also opens the possibility for high-throughput screening of enzymes with increased selectivity for NADH. In our cases, similar XR levels were measured (data not shown). However, in future screening, an enzyme with lower specificity for NADH, but expressed at a higher level and giving a higher activity/per cell for NADH oxidation will lead to a lower FI. This limitation in the screening will, however, occur in any method based on the NADH pool and not only in the FI-based one. Another element that can be further considered when using a GFP-based reporter system for screening or analysis of the NAD(H) cofactor imbalance is the half-live of the chosen protein. The yEGFP3 protein used in the present study has been reported to have a half-life of about 7.5 hours (Mateus and Avery [2000]), and indeed the intracellular GFP level did not respond rapidly to dynamic changes in external conditions. This implies that rapid changes in NAD(H) cofactor balancing may not be monitored with the developed system. However alternative GFP variants with shorter half-life, such as the GFP fused with a fragment of the CLN2 (yeast G1 cyclin) protein having a half-life of 30 minutes (Mateus and Avery [2000]), could then be used instead.

Lately, much attention has been drawn in using FACS for screening large genetic space (Dietrich et al. [2010]) and the technique has already been applied for the screening of NADPH-dependent alcohol dehydrogenases in *E. coli* (Siedler et al. [2014]). However, the combination of a NADH biosensor and single-cell analysis has, to our knowledge, not yet been implemented in *S. cerevisiae*. Our tool offers the first single-cell high-throughput NAD(H) cofactor balance sensor in *S. cerevisiae*. In contrast to previously reported methods where extraction of intracellular NAD(H) metabolites was performed (Canelas et al. [2008]), the method presented in this work is faster and allows for analysis with single-cell level resolution. Therefore the constructed tool offers possible applications in the selection of new NADH-dependent enzymes based on single cell sorting. Additionally it could be applied in monitoring the impact of various metabolic engineering strategies on the cytosolic NAD(H) cofactor balance of the cell.

## Competing interests

The authors declare that they have no competing interests.

## Authors’ contribution

JDK, MC and MGG contributed equally in the design of the study. JDK performed the experimental work and wrote the manuscript. MC and MGG helped in drafting the manuscript. All authors have read and approved the submission of the manuscript.
